# Macular Ganglion Cell Layer Thickness after Macula-Off Rhegmatogenous Retinal Detachment Repair: Scleral Buckling versus Pars Plana Vitrectomy

**DOI:** 10.3390/jcm9051411

**Published:** 2020-05-10

**Authors:** Magda Gharbiya, Giuseppe Maria Albanese, Andrea Maria Plateroti, Michela Marcelli, Marco Marenco, Alessandro Lambiase

**Affiliations:** Ophthalmology Unit, Department of Sense Organs, Sapienza University of Rome, Umberto I University Hospital, 00161 Rome, Italy; giuseppemaria.albanese@uniroma1.it (G.M.A.); andrea.plateroti@uniroma1.it (A.M.P.); michela.marcelli@uniroma1.it (M.M.); marco.marenco@uniroma1.it (M.M.); alessandro.lambiase@uniroma1.it (A.L.)

**Keywords:** optical coherence tomography, segmentation, retinal imaging analysis, ganglion cell layer, inner plexiform layer, retinal detachment, scleral buckling, pars plana vitrectomy

## Abstract

(1) Background: We evaluated macular ganglion cell layer–inner plexiform layer (GCL-IPL) thickness in patients with primary macula-off rhegmatogenous retinal detachment (RRD) treated with scleral buckling (SB) or pars plana vitrectomy (PPV) using spectral domain optical coherence tomography (SD-OCT). (2) Methods: In this retrospective, observational study, we reviewed the medical records of patients undergoing SB or PPV surgery for macula-off RRD. SD-OCT was performed at three and 12 months after surgery. The central and parafoveal GCL-IPL thicknesses in treated eyes were compared with those of healthy fellow eyes. OCT measurements between the SB and PPV group were also compared using the analysis of covariance. (3) Results: Seventy-one eyes of 71 patients with a mean age of 61.2 ± 11.7 years were included. The parafoveal GCL-IPL thickness of the PPV group was significantly reduced, with respect to fellow eyes, at three and 12 months (*p* < 0.01). After adjusting for age, axial length, spherical equivalent, RD extent, preoperative intraretinal cysts, duration of symptoms and postoperative IOP, the parafoveal GCL-IPL thickness in the PPV group was significantly reduced with respect to the SB group, both at three and 12 months (F = 11.45, *p* = 0.001 and F = 12.37, *p* = 0.001, respectively). (4) Conclusions: In conclusion, the GCL-IPL is reduced in thickness in eyes with macula-off RRD treated with vitrectomy and is significantly thinner compared to eyes undergoing scleral buckling surgery.

## 1. Introduction

The annual incidence of primary rhegmatogenous retinal detachment (RRD) has been estimated between 6.3 and 17.9 per 100,000 populations with significant geographical variation and a mean proportion of bilateral cases of 7.3% [1]. Primary RRD may be managed using two main surgical procedures, namely scleral buckling (SB) and pars plana vitrectomy (PPV). A combination of both methods is sometimes used in selected and most complicated cases. The choice of method and its technical specificities varies considerably among surgeons and center, partly because to date there is still no strong certainty evidence of significant differences in both the functional and anatomical outcome between the two procedures. In the literature, several studies have investigated the role of surgical procedure on visual outcomes after RRD repair, sometimes with conflicting results [2,3]. A recent metanalysis that included 10 randomized controlled trials (1307 eyes of 1307 patients), comparing SB and PPV for primary RRD repair, found no evidence of any important difference in postoperative visual acuity between the procedures with a mean difference of 0.00 logMAR (95% CI −0.09 to 0.10) and fewer re-detachments in PPV-treated eyes (21% vs. 28%, 95% CI 0.59 to 0.96, *p* = 0.02) by three to 36 months [4]. Nevertheless, in a clinical setting, despite anatomically successful RRD repair, even if the macula has not been involved, patients may show incomplete functional recovery or visual complaints in the absence of manifest retinal alterations. 

The recent advent of spectral-domain optical coherence tomography (SD-OCT) has allowed the detection of microstructural changes in the retinal layers of reattached retina. Previous studies, using SD-OCT in this setting, mainly focused on the outer retinal changes [5,6], whereas only few studies have examined the effect of RRD repair on inner retinal layers with unclear results: the inner retinal layers’ thickness was unchanged in some studies [7,8,9], while others reported a thickness decrease [10,11]. 

In the present paper, using SD-OCT automated segmentation, we evaluated macular ganglion cell layer–inner plexiform layer (GCL-IPL) thickness in patients with primary macula-off RRD treated with scleral buckling or pars plana vitrectomy compared to non-treated fellow eyes.

## 2. Materials and Methods

The present investigation was an institutional retrospective study with fellow-eye comparison. The study was approved by the ethical board of the Sapienza University of Rome and was conducted in accordance with the tenets of the Declaration of Helsinki. All patients gave written informed consent to the study. We reviewed the medical records of patients who underwent anatomically successful repair for primary RRD treated with SB or PPV performed by a single experienced retinal surgeon (MG) at the Ophthalmology Unit of the Policlinico Umberto I University Hospital of Rome, from January 2013 to January 2018. 

The inclusion criteria were: (1) primary macula-off RRD successfully repaired by a single uncomplicated SB or PPV surgical procedure, and (2) a follow-up at least 12 months after surgery. Only patients with clear ocular media were included in this study (nuclear color/opalescence, cortical, or posterior subcapsular lens opacity < 1) according to the Lens Opacities Classification System III [12].

Exclusion criteria in the treated and fellow eye were as follows: (1) macular pathologies such as age-related macular degeneration or any disease affecting the vitreo-macular interface, e.g., epiretinal membrane and macular hole, (2) presence of glaucoma or intraocular pressure above 18 mmHg during follow-up, (3) diabetes or history of retinal vascular occlusion, (4) previous intraocular surgery other than uncomplicated cataract surgery, (5) any further surgery during the follow-up period including cataract surgery, (6) history of uveitis or any ocular pathology, other than non-complicated RRD, (7) spherical equivalent beyond three diopters and axial length above 26.5 mm or below 22 mm, (8) any proliferative vitreo-retinopathy, and (9) low-quality (<25 units) or unreliable OCT images. 

All patients in the SB group underwent encirclage with a 240-silicone band, external subretinal fluid drainage and cryotherapy application to the retinal break(s). A circumferential silicone scleral explant was finally positioned to close the break(s).

A 23-gauge vitrectomy was performed in all cases of the PPV group, using the EVA Phaco-Vitrectomy System (D.O.R.C. International, Zuidland, the Nederlands). After core vitrectomy, following creation or confirmation of posterior vitreous detachment, peripheral vitrectomy and vitreous base shaving was performed with scleral indentation using a non-contact wide-viewing system. Endophotocoagulation was applied to all the retinal breaks after perfluorocarbon liquid (PFCL)/air exchange. Air/gas exchange with 12% C3F8 (MICROMED s.r.l., Rome, Italy) was performed at completion of the procedure. In phakic eyes standard phacoemulsification and intraocular lens implantation was performed before vitrectomy. In the clinical setting of our tertiary center the decision to perform SB or PPV depends mainly on the presence of media opacity as well as on the number and location of retinal break(s).

As part of a standardized protocol, all patients underwent a complete ophthalmological evaluation including ocular biometry (IOL Master 500, Carl Zeiss Meditec, Dublin, CA, USA) and SD-OCT, both before and after surgery at three and 12 months.

Preoperative data collection included a complete medical and ophthalmic history, best-corrected visual acuity (BCVA), axial length, spherical equivalent, lens status, intraocular pressure (IOP) and time from onset of symptoms to surgery. The characteristics of the RDs and the presence of macular involvement were evaluated by binocular indirect ophthalmoscopy and OCT. BCVA was measured using the standardized, 70-letter Early Treatment Diabetic Retinopathy Study (ETDRS) chart (Chart ‘R’, Precision Vision, La Salle, IL, USA) at a 4-m distance. For statistical analysis, both preoperative and postoperative BCVA was analyzed on a logarithm of the minimal angle of resolution scale (logMAR). LogMAR values of 1.85 and 2.30 were assigned for counting fingers and hand motions vision at 2 feet, respectively.

**Figure 1:**
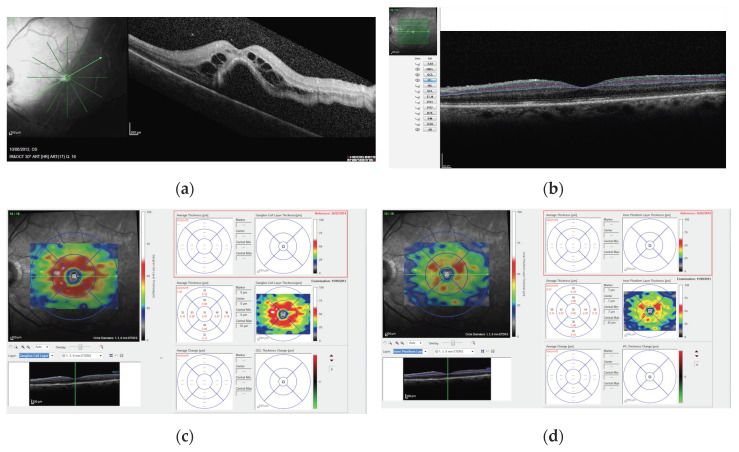
(**a**) Preoperative infrared and SD-OCT B scan images of a patient with macula-off RRD undergoing PPV surgery; (**b**) postoperative ganglion cell (GCL) and inner plexiform (IPL) layers automated segmentation, where the turquoise line represents the boundary between the retinal nerve fiber layer and the GCL, the purple line represents the boundary between the GCL and IPL, and the blue line represents the boundary between the IPL and the inner nuclear layer; postoperative retinal thickness maps showing average GCL (**c**) and IPL (**d**) thickness in the ETDRS grid. The magnification scale of all OCT images is 200 microns.

SD-OCT (Spectralis OCT Family Acquisition Module, V 6.0.11.0 Heidelberg Engineering, Heidelberg, Germany) records included near infrared images and macular scans with the raster 20° × 20°, 19-line scan protocol with an interval between scans of 240 μm and 100 frames averaged for each scan. Active eye-tracking (TrueTrack Heidelberg Engineering, Heidelberg, Germany) and automated follow-up scan (AutoRescan Heidelberg Engineering, Heidelberg, Germany) were used to enable point-to-point correspondence between consecutive follow-up scans. The automated segmentation protocol of the Spectralis OCT (Heidelberg Eye Explorer V1.9.10.0, Heidelberg, Germany) was used to measure retinal thicknesses of the GCL and IPL layers according to the OCT Consensus guidelines for landmarks in OCT [13]. Two experienced investigators (M.G. and M.M.) evaluated the automated segmentation and manually corrected for any misalignment. The average measurements were recorded with the ETDRS macular grid at 1 and 3. This grid consists of a central subfield (1 mm in diameter), the inner ring (1 mm in width and 3 mm in diameter, with four sectors), and the outer ring (1.5 mm in width and 6 mm in diameter, with four sectors). The 3-mm GCL and IPL thickness was calculated by averaging the four sector values of the inner ring without including the central 1-mm subfield (Figure 1).

Statistical analysis was performed with the SPSS for windows (V 17.0; SPSS, Inc., Chicago, IL, USA). Normal distribution of data was analyzed by the Kolmogorov–Smirnov test. Parametric variables were compared using the unpaired *t*-test with Levene’s correction. Nonparametric distributed values were analyzed by the Mann–Whitney rank-sum test. Categorical variables were compared using Fisher’s exact test. Bivariate relationships were evaluated by the Spearman coefficient or the Pearson analysis, as appropriate. OCT measurements between treated and fellow eyes were compared using the paired *t*-test or the Wilcoxon’s signed ranks test, as appropriate. OCT measurements between the SB and PPV group were compared using the analysis of covariance (ANCOVA) including age, axial length, spherical equivalent, RD extent, preoperative intraretinal cysts, duration of symptoms and postoperative IOP, as covariates.

*p*-values of <0.05 were considered as statistically significant. Data are reported as mean values ± standard deviation.

## 3. Results

We reviewed the medical records of 367 patients with simple primary RRD that were treated between January 2013 and January 2018, of whom 195 patients underwent SB surgery and 172 PPV. Retinal re-detachment was observed in 35 eyes in the SB group and 25 eyes in the PPV group. The retinal re-detachment rate at 12 months was 17.9% and 14.5% in the SB and PPV group, respectively (*p* > 0.05).

A total of 71 eyes of 71 patients (43 men, 28 women) with a mean age of 61.2 ± 11.7 years (range, 36 to 89) were finally included to address the purposes of the present study. Forty eyes underwent SB surgery and 31 eyes underwent PPV. The mean duration of visual symptoms was 7.1 ± 5.5 days (range, 2 to 34). At baseline, there were no significant differences in mean age and sex distribution between groups (*p* > 0.05). Compared to eyes undergoing SB surgery, in the PPV group there was a higher proportion of patients with a retinal detachment involving more than two quadrants (*p* = 0.03). No further significant differences were observed among the recoded preoperative characteristics. The baseline clinical characteristics of treated and fellow eyes in the SB and the PPV group are shown in Table 1.

The macular 3-mm GCL-IPL thickness of the PPV group was significantly reduced with respect to fellow eyes, at three and 12 months (*p* = 0.005 and *p* = 0.003, respectively), whereas no significant difference in the macular GCL-IPL thickness was found between treated and fellow eyes in the SB group. There were no longitudinal changes in macular GCL-IPL thickness in both groups (*p* > 0.05).

During the 12-month follow-up period, there were no differences in postoperative IOP between the SB and PPV group (*p* > 0.05). Table 2 shows the postoperative results of OCT segmentation analysis and visual outcome.

Further, after adjusting for age, axial length, spherical equivalent, RD extent, preoperative intraretinal cysts, duration of symptoms and postoperative IOP, the macular 3-mm GCL-IPL thickness in the PPV group was significantly reduced with respect to the SB group, both at three and 12 months (F = 11.45, *p* = 0.001 and F = 12.37, *p* = 0.001, respectively).

There were no significant differences in baseline BCVA between the SB and the PPV group (*p* > 0.05). Compared to baseline, BCVA improved in both groups up to 12 months after surgery (*p* < 0.0001). In addition, there were no difference between groups in postoperative BCVA outcome both at three and 12 months (*p* > 0.05).

**Figure 2:**
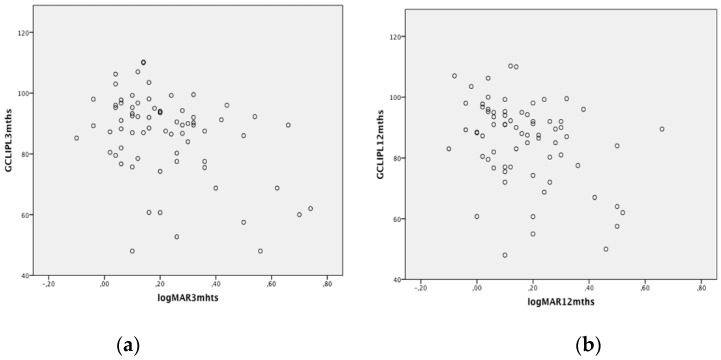
Scatterplots showing the correlation between logMAR visual acuity outcome and the macular 3-mm GCL-IPL thickness at 3 (**a**) and 12 months (**b**) after surgery.

Finally, in the overall sample of operated eyes, we found a significant correlation between logMAR BCVA outcome and the macular 3-mm GCL-IPL thickness (*r* = −0.393, *p* = 0.001 and *r* = −0.337, *p* = 0.004, at three and 12 months, respectively), (Figure 2). Postoperative logMAR BCVA was also significantly correlated with the presence of preoperative intraretinal cystic changes (*r* = 0.299, *p* = 0.02 and *r* = 0.278, *p* = 0.03 at three and 12 months, respectively).

## 4. Discussion

The present study shows that, compared to fellow eyes, the macular 3-mm GCL-IPL thickness is reduced in PPV but not in SB and that, with respect to eyes treated with SB, there is a significant thinning of the macular 3-mm GCL-IPL layer in eyes treated with PPV. Correlation analysis shows a significant correlation between BCVA outcome and the macular 3-mm GCL-IPL thickness.

Experimental models have shown that retinal detachment primarily affects the outer retinal layers with minimal and only late change in the inner retinal layers [14,15,16,17,18,19]. In agreement with these findings, we did not observe any significant change in the GCL-IPL thickness after retinal reattachment in eyes undergoing scleral buckling probably also for the short onset duration of detachment (mean duration of presumed macular detachment, 7.5 ± 6.2 days). By contrast, we found a sustained GCL-IPL thinning in eyes treated with PPV up to 12 months after surgery.

In a previous study that analyzed retinal layers thickness after RRD repair, Kim et al. failed to demonstrate significant changes in the inner retinal layers [7]. However, the results of this study refer to a mixed sample including 14 patients treated with SB and 14 treated with PPV. In addition, only four thickness measurement points were taken manually at 2800 and 3000 microns from the center and no data are given accounting for intra-observer variability. Lee et al., in a recent study evaluating retinal layers’ thickness changes in 31 eyes with macula-on RRD that underwent PPV with gas injection, did not observe any significant change of the 1-mm GCL and IPL thickness [8]. Similarly, we found no significant difference in the 1-mm GCL-IPL thickness between eyes that underwent PPV and their fellows. However, while they have evaluated inner retinal layers’ thickness only in the 1-mm subfield, we evaluated it also in the 3-mm ETDRS area. While no significant difference was detected between the 1-mm GCL-IPL thickness in PPV eyes and their fellows, our results revealed a significant decrease in the 3-mm diameter parafoveal area. The inability to detect change in GCL and IPL thickness in the central 1-mm area may be linked to the anatomical structure of the macula which shows gradual thinning of the inner retinal layers as they approach the fovea to terminate in the central foveal avascular zone. Further, our findings agree with those of a previous prospective study using SD-OCT automated retinal segmentation [11], which revealed a significant reduction of the GCL-IPL thickness in a cohort of 33 patients with macula-off RD treated with PPV and gas tamponade.

Overall, the results of the present study suggest that GCL-IPL thinning is a direct result of PPV surgery. In a previous prospective study, Koutsandra et al. evaluated visual field defects in RD cases treated with SB versus PPV and gas injection, they found that after surgery the retinal functionality of the preoperative attached areas was worse in the PPV group compared to the SB group [20]. In agreement with this study, we postulate that, since RD primarily affect the photoreceptor layer, the GCL-IPL thinning found in our cases treated with PPV may be related to minor additional traumatizing factors related to PPV itself. Variation of ocular perfusion pressure and/or relative retinal ischemia due to elevated IOP during vitrectomy may possibly act as damaging factors on the GCL-IPL layers. In this setting, our finding of parafoveal GCL-IPL thinning sparing the central 1-mm subfield, where the foveal avascular zone lies, seems to corroborate this hypothesis. Indeed, the inner retinal layers are supplied by the retinal vascular network, whereas the outer retina is prevalently supplied by the choroidal circulation. Several additional factors have been described that may potentially have damaging effects on the inner retinal layers during vitrectomy such as retinal dehydration caused by air infusion, use of perfluorocarbon liquid, gas toxicity, and mechanical trauma to the optic nerve [21,22,23,24]. A last but not minor potential issue in this context may be the removal of the vitreous during PPV. The vitreous is an essential storage area of nutrients and metabolite for the retina that it receives from synthesis within the non-pigmented ciliary epithelium and retinal pigment epithelium. While the functions of many vitreous proteins are still unknown, there is evidence that some of them provide antioxidative and neuroprotective functions to the surrounding ocular tissue [25,26]. Deficient vitreous antioxidant capacity and associated concurrent increase in intravitreal reactive oxygen species (ROS) and proteolytic enzymes may contribute to retinal damage [27,28,29,30]. Further, in an experimental RD model in rats, Kawano et al. demonstrated that the cytotoxic and inflammatory effects of extracellular histones released during RD were neutralized in vitro in the presence of vitreous. This inhibitory activity was attributed to the trapping property of hyaluronan present in the vitreous that prevented the spread of histones and retinal cells’ damage [31]. Recently, Ankamah et al. postulated that reattachment of the neurosensory retina following RD repair can trigger hyperoxia and increased free radical generation, culminating in reperfusion injury similar to CNS strokes [32]. Vitreous antioxidant activity could mitigate the reperfusion injury effects, unless depleted [33].

In contrast to our results, Hong et al., in a recent study on 31 RD patients undergoing PPV, found that the thickness of the parafoveal GCL-IPL layer in the treated eyes did not differ significantly from those of the fellow eyes [9]. However, in this study, the mean age of patients was considerably lower compared to our cohort (53.3 ± 14.4 vs. 62.0 ± 11.6 years, respectively). It could be postulated that older patients, who usually have systemic comorbidities, might be more sensitive to the abovementioned damaging factors potentially associated with vitrectomy. Although, in our sample we did not find any correlation between age and postoperative GCL-IPL thickness, further investigations on a larger study population are warranted in this context that will analyze the relation between inner retinal layer thickness change after vitrectomy and age as well as concomitant systemic diseases.

There was no significant difference in visual outcome between SB- and PPV-treated eyes; however, we found a tendency of worse postoperative VA in the PPV group. This finding may be partly explained by the mechanisms mentioned above related to vitrectomy itself and/or to vitreous removal with loss of its protective and antioxidant function. However, we can certainly not exclude that our results could have been confounded by selection bias related to differences in the preoperative characteristics of RDs. In the clinical setting of our center, the decision to perform SB or PPV depends mainly on the presence of media opacity and the number and location of retinal breaks. Therefore, compared to SB eyes, PPV-treated eyes probably had a higher rate of multiple breaks and more posterior breaks. Further, in the PPV group there was a higher proportion of RD involving more than two quadrants. Interestingly, we found an overall significant correlation between VA outcome and the macular 3-mm GCL-IPL thickness up to 12 months after RRD repair. While many studies have analyzed the relation of visual outcome after RRD repair with the outer retina, few have assessed the inner retinal layers. Similar to our findings, a previous study [34] evaluating the relation between retinal thickness in parafoveal subfields and VA after vitrectomy, reported a significant correlation between inner retinal layer thickness and postoperative VA, concluding that structural changes in both the outer and the inner retinal layers may affect visual outcome after macula-off RRD repair.

Overall, our results seem to show that, with respect to SB surgery, vitrectomy may affect the GCL-IPL layer. Albeit, the macular GCL-IPL thinning in PPV eyes does not seem to significantly influence the final visual acuity, it may underpin a functional involvement at the level of retinal sensitivity as observed previously by static automated perimetry [20]. Thus, in this context, the prevention of ganglion cells’ damage might be a good target for future neuroprotective therapies to improve visual function after RRD repair.

The strengths of this work are the application of strict inclusion and exclusion criteria and the use of the automated segmentation software of the Spectralis SD-OCT that showed excellent repeatability and reproducibility [35]. The shortcomings are the retrospective non-randomized design and the relatively small sample size. Further, our study cannot definitely rule out the possible effect of retinal detachment on the GCL-IPL thinning as we evaluated macula-off RRD only, as well as we cannot clarify the cause-effect relationship of GCL-IPL thinning associated with PPV and visual function, and this would require further research.

In conclusion, we demonstrated GCL-IPL thinning in eyes with macula-off RRD treated with vitrectomy while no changes were observed in those undergoing scleral buckling surgery. We postulated that these findings could be attributed to additional traumatizing factors related to vitrectomy. These results need to be confirmed in future investigations with a larger study population.

## Figures and Tables

**Table 1:** Baseline clinical characteristics of treated and fellow eyes in the SB and the PPV group.

	Scleral Buckling		Pars Plana Vitrectomy		*p*-Value
	Treated Eyes	Fellow Eyes	Treated Eyes	Fellow Eyes	
Age, years	60.5 ± 11.9		62.0 ± 11.6		0.5 *
Sex (male/female)	24/16		19/12		0.5 §
Axial length (mm)	24.6 ± 0.7	24.5 ± 1.0	24.3 ± 0.8	24.1 ± 0.9	0.1 **
Spherical equivalent (dioptres)	−1.1 ± 1.3	−0.9 ± 1.4	−0.8 ± 1.5	−0.6 ± 1.2	0.4 **
Lens status (phakic/pseudophakic)	21/19	24/16	20/11	21/10	0.3 §
Duration of symptoms (days)	7.5 ± 6.2		6.6 ± 4.4		0.5 **
Preoperative BCVA (logMAR)	1.28 ± 0.62		1.32 ± 0.67		0.8 **
Retinal detachment extent >2 quadrants	16/40		21/31		0.03 §
Cystic changes in the retinal layers	32/40		26/31		0.8 §
IOP (mmHg)	12.1 ± 2.3	13.0 ± 1.6	11.7 ± 2.1	13.3 ± 1.5	0.5 *

* Unpaired *t*-test. ** Mann–Whitney rank-sum test. § Fisher’s exact test.

**Table 2:** Postoperative results of OCT segmentation analysis and visual outcome of treated and fellow eyes in the SB and the PPV group.

	Scleral Buckling		*p*-Value *	Pars Plana Vitrectomy		*p*-Value *	*p*-Value
	Treated Eye	Fellow Eye		Treated Eye	Fellow Eye		
1-mm GCL-IPL at 3 months (microns)	42.1 ± 10.6	40.0 ± 8.4	0.8	41.2 ± 11.4	40.7 ± 8.5	0.6	F = 1.17 *p* = 0.9 **
3-mm GCL-IPL at 3 months (microns)	91.8 ± 10.2	91.2 ± 8.4	0.3	79.5 ± 12.6	91.9 ± 8.7	0.005	F = 11.45 *p* = 0.001 **
1-mm GCL-IPL at 12 months (microns)	41.4 ± 11.0	41.0 ± 9.8	0.9	40.9 ± 11.7	41.2 ± 8.5	0.7	F = 1.65 *p* = 0.4 **
3-mm GCL-IPL at 12 months (microns)	92.2 ± 10.2	92.0 ± 8.8	0.5	77.7 ± 12.9	92.1 ± 8.8	0.003	F = 12.37 *p* = 0.001 **
logMAR BCVA at 3 months	0.19 ± 0.14			0.25 ± 0.17			*p* = 0.1 °
logMAR BCVA at 12 months	0.14 ± 0.14			0.21 ± 0.19			*p* = 0.08 °
IOP at 3 months (mmHg)	13.2 ± 1.8			13.6 ± 1.5			0.3 §
IOP at 12 months (mmHg)	13.3 ± 1.9			13.9 ± 1.6			0.2 §

* Paired *t*-test. ** Analysis of covariance including age, axial length, spherical equivalent, RD extent, intraretinal cysts, duration of symptoms and postoperative IOP, as covariates. ° Mann–Whitney rank-sum test. § Unpaired *t*-test.
